# Detection of selection signatures in indigenous African cattle reveals genomic footprints of adaptation, production and temperament traits

**DOI:** 10.1007/s00335-026-10193-9

**Published:** 2026-01-28

**Authors:** Rodney Okwasiimire, Donald R. Kugonza, Junxin Gao, Melak Weldenegodguad, Mahlako L. Makgahlela, Nasser Ghanem, Catarina Ginja, Richard P. M. A. Crooijmans, Juha Kantanen, Pekka Uimari, Kisun Pokharel

**Affiliations:** 1 Department of Agricultural Sciences, University of Helsinki, Helsinki, Finland; 2 Natural Resources Institute Finland, Jokioinen, Finland; 3 Department of Animal and Range Sciences, College of Agricultural and Environmental Sciences, Makerere University, Kampala, Uganda; 4 Animal Breeding and Genomics, Wageningen University and Research, Wageningen, The Netherlands; 5 Natural Resources Institute Finland, Helsinki, Finland; 6 Animal Breeding and Genetics, Agricultural Research Council, Pretoria, South Africa; 7 Department of Animal, Wildlife and Grassland Sciences, University of the Free State, Bloemfontein, South Africa; 8 Department of Animal Production, Cairo University, Cairo, Egypt; 9 CIBIO, Centro de Investigação em Biodiversidade e Recursos Genéticos, InBIO Laboratório Associado, Campus de Vairão, Universidade do Porto, Vairão, Portugal; 10 BIOPOLIS, Program in Genomics, Biodiversity and Land Planning, CIBIO, Campus de Vairão, Vairão, Portugal

**Keywords:** Native cattle, Whole genome sequencing, Raised accuracy in sweep detection (RAiSD), Runs of homozygosity (ROH), Adaptation, Genomic selection

## Abstract

Indigenous cattle account for approximately 80% of Uganda’s cattle population. These animals are well adapted to the country’s ten agroecological zones and are mainly kept under pastoral and agropastoral systems. Unlike commercial breeds, they thrive on low-quality feeds, while tolerating major tropical diseases and parasites including tsetse flies, ticks, and vector-borne infections. Whole-genome sequence (WGS) analysis offers opportunities to uncover genomic regions underlying these adaptations and to trace the genetic footprints of long-term breeding decisions taken by cattle keepers. In this study, WGS data from 95 animals representing six indigenous cattle populations (Ankole, Karamojong, Nganda10, Nganda17, Nkedi, and Ntuku) were analyzed to identify genomic regions under putative selection. Two complementary approaches were applied: enumeration of the µ-statistic in RAiSD and runs of homozygosity (ROH) analysis. RAiSD identified population-level signals, while conserved ROH regions were defined using breed-specific SNP-incidence thresholds. The two methods identified 803 and 49 candidate genes respectively. The top genes identified included *SLC37A1* (BTA1), *CHCHD3* (BTA4), and *RAB3GAP1* (BTA2) detected by RAiSD, and *IL26* (BTA5), *FBXL7* (BTA20), and *HSPA9* (BTA7) contained in ROH. Furthermore, the regions harbored 107 novel genes (92 detected by RAiSD and 15 by ROH), corresponding to 255 quantitative trait loci. The identified genes under putative selection are associated with economically important traits including adaptation to tropical environments, resistance to parasites and diseases, and other farmer-preferred characteristics. These findings provide insights into the genetic basis of adaptation, selection and production in Ugandan indigenous cattle, supporting conservation and breeding strategies to enhance resilience and productivity.

**Supplementary Information:**

The online version contains supplementary material available at 10.1007/s00335-026-10193-9.

## Introduction

African indigenous cattle are adapted to tropical environmental conditions which are characterized by high temperatures, seasonal drought, and exposure to a diverse plethora of parasites, vectors and diseases. The animals are typically managed extensively with minimal inputs under pastoral and agropastoral livestock systems, where they are moved seasonally in search of water and pasture (Kabi et al. 2016).

In Uganda, indigenous cattle fall into three major groups: the Sanga (Ankole, Kigezi, Nyoro, Ntuku), the Zenga (Nganda), and East African Shorthorn Zebu (EASZ). These grouping names reflect historical classifications, although the specific breed names used vary across the African continent. The EASZ are further divided into two subcategories: small and large, based on body size (Mwacharo et al. 2006). The large EASZ (Karamojong cattle) are reared in the semi-arid parts of Northeastern Uganda, while the small EASZ (Lugware, Teso, Usuk, Kyoga, Serere, and Nkedi cattle) are found in the moist parts of the eastern and northern regions (Rege 1999). The Sanga and Zenga have traditionally been managed in the western and central regions of the country, respectively.

Uganda has up to ten agroecological zones (defined as areas with analogous land use practices and climate) (Masaba et al. 2024). Consequently, factors such as vegetation cover (and therefore available pasture), water availability, grazing land, parasites, vectors, diseases, and the specific use of animals differ among cattle populations. Although indigenous cattle in Uganda are not subjected to intensive, structured breeding programs like commercial cosmopolitan breeds, they experience low intensity, but consistent artificial selection imposed by management practices of cattle keepers. In a recent study (Ekou and Ocaido 2025), respondents mentioned draught power (25.0%), and savings/investment (36.7%) among the main reasons for keeping cattle. This suggests that herders make breeding decisions such as which animals to retain, exchange, or cull, based on performance and economic value. Over generations, these culturally and economically guided choices have contributed to gradual artificial selection, shaping the present-day indigenous breeds alongside environmental pressures.

Indigenous cattle possess unique traits that have enabled them to adapt to the challenging tropical environment. These include lower heat production, reduced metabolic rate, ability to consume low-quality (and often quantity) feed, reduced water requirements and superior tolerance to parasites, vectors and vector-borne infections (Wilson 2009). Through natural selection, animals with such well-developed traits likely had higher fitness to the tropics than those with inferior abilities. In contrast, the multi-generational decisions made by traditional farmers about which animals were permitted to sire offspring, and which were culled likely constituted an early form of artificial selection, shaped primarily by farmers’ preferences for specific phenotypic traits. Examples of these traits are product yield and quality, adaptive features, and physical characteristics (Rege 2003). Other features that have been studied in this regard include morphological characteristics such as coat color, size and shape of horns (Kabi et al. 2015; Kugonza et al. 2011; Masaba et al. 2024; Ndumu et al. 2008a, b), as well as behavior and docility (Wurzinger et al. 2008).

Both natural and artificial selection leave distinct patterns of sequences in certain regions of the genome. These regions often hold functionally significant variants and are defined as selective sweeps or signatures of selection. Identification of such regions provides insights into the selection and adaptation history of livestock (Pariset et al. 2009). In addition, it defines animal conservation ambitions and informs the formulation of breeding programs for the improvement of economically important traits for enhanced production efficiency.

Various tests for selection signatures that compare genomic diversity and differentiation within (intra) populations or between (inter) populations, have been reviewed (Gouveia et al. 2014; Saravanan et al. 2020). These often evaluate single SNP metrics such as linkage disequilibrium (LD), runs of homozygosity (ROH), site frequency spectrum (SFS), reduced variation and genetic differentiation using haplotypes (Saravanan et al. 2020). Some other methods identify signatures of selection through enumeration of multiple SNP metrics. One example is RAiSD (Raised Accuracy in Sweep Detection) (Alachiotis and Pavlidis 2018), which computes the µ-statistic, a composite score of multiple genomic features including changes of the SFS, LD, and the genetic diversity along a genomic region.

Selection signatures in several indigenous African cattle breeds have been investigated in recent years. Notable examples include studies on environmental adaptation in Sheko cattle (Bahbahani et al. 2018a, b), East African Shorthorn Zebu (Bahbahani et al. 2015, 2017), Butana and Kenana cattle of Sudan (Bahbahani et al. 2018a, b), thermotolerance in selected African cattle breeds (Taye et al. 2017a). Other studies include environmental adaptation in African cattle (Kim et al. 2020), trypanotolerance in N’Dama cattle (Kim et al. 2017a) and disease resistance and artificial selection in sub-Saharan African cattle (Kim et al. 2017b). Although, the Ankole breed from Uganda has been included in some of these studies, it has not been comprehensively explored, with only one exclusive study focused on genes linked to beef quality of this breed (Taye et al. 2017b). Consequently, the signatures of selection in Ankole and other native Ugandan cattle populations have remained largely unexamined.

The present study aims to fill this gap through implementing two approaches to detect genomic regions under putative selection at the population (RAiSD) and individual (conserved ROH analysis) levels. RAiSD was adopted because it captures multiple signals of selection. The ROH approach was adopted based on the understanding that deleterious mutations are typically purged from the gene pool before reaching detectable frequencies, leaving advantageous mutations as conserved genomic segments characterized by reduced variation (Vitti et al. 2013). ROHs can also provide insights into breed population history, as events such as population bottlenecks, selection pressures, and breeding practices may leave characteristic imprints on the segments (Purfield et al. 2012). RAiSD has previously been used in studies on Ladakhi cattle (Koloi et al. 2025), Simmental and Red Angus cattle (Rowan et al. 2024), and domestic reindeer (*Rangifer tarandus*) (Pokharel et al. 2023). Likewise, ROH analysis has been applied to detect signatures of selection in Indian (Nayak et al. 2023, 2025; Rajawat et al. 2022), South and North American (Garduño et al., 2024; Rocha et al. 2023), Chinese (Zhao et al. 2021) and Korean cattle (Ju et al., 2025).

The analyzed indigenous cattle populations included: two Sanga (Ankole and Ntuku), two Zenga (Nganda10 and Nganda17), one Large EASZ (Karamojong) and one Small EASZ (Nkedi). Two populations of the Zenga group were considered based on a previous study that identified a sub-population of Nganda cattle (Okwasiimire et al. 2025).

## Materials and methods

### Data Preparation

**Fig. 1:**
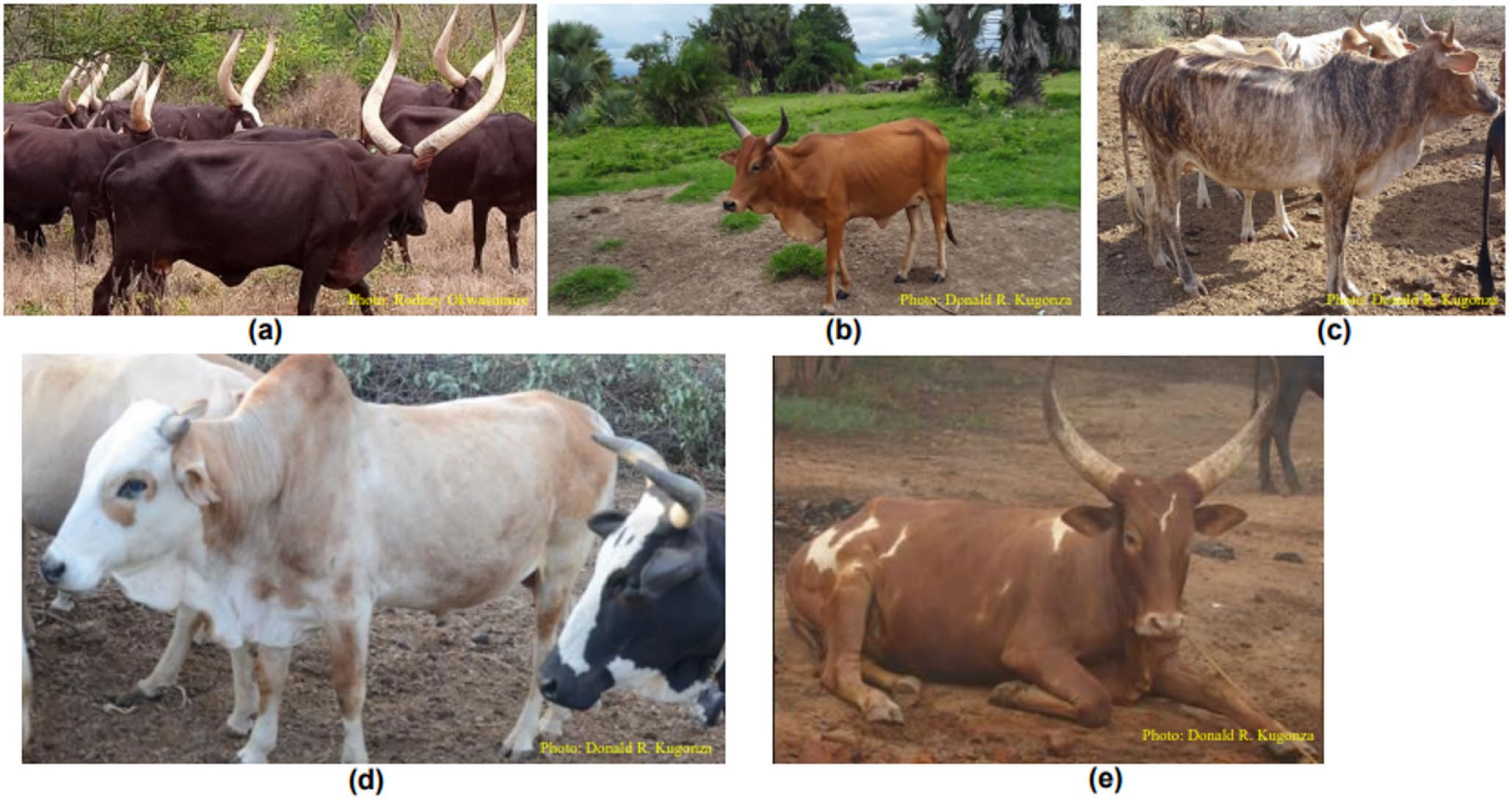
Photographs of the cattle breeds included in this study. **a** Ankole breed of the Sanga type, **b** Ntuku breed of the Sanga type, **c** Nkedi breed of the Small East African Shorthorn Zebu type, **d** Karamojong breed of the Large East African Shorthorn Zebu type and **e** Nganda breed of the Zenga type (Okwasiimire et al. 2025)

A total of 95 animals including the Ankole (*n* = 19), Karamojong (*n* = 11), Nganda10 (*n* = 10), Nganda17 (*n* = 17), Nkedi (*n* = 19) and Ntuku (*n* = 19) were drawn from six native cattle populations (Fig. 1) of Uganda. The Nganda10 and Nganda17 populations both belong to the Nganda breed but have been previously described as distinct sub-populations (Okwasiimire et al. 2025). Genomic DNA was extracted from venous blood following the salting-out procedure (Miller et al. 1988), and quantified with a UV/Vis spectrophotometer (Biochrom Ltd., Cambridge, UK). Paired-end sequencing (PE150) was done on an Illumina NovaSeq 6000 platform (Illumina Inc., San Diego, CA, USA) using single-indexed genomic libraries. Quality checks of the raw FASTQ files were conducted with FastQC v0.11.9 (Andrews 2010), and results were compiled with MultiQC v1.19 (Ewels et al. 2016). Adapter sequences and low-quality reads were trimmed with Trimmomatic v0.39 (Bolger et al. 2014) in default mode.

Read alignment and variant processing were performed according to the Genome Analysis Toolkit (GATK) v4.6.0.0 best practices (Van Der Auwera et al. 2013). High-quality reads were aligned to the *Bos taurus* ARS-UCD1.3 genome assembly (Ensembl release 112) using BWA-MEM v0.7.17 (H. Li and Durbin 2010). SAMtools v1.18 (Li et al. 2009) was used to convert SAM to BAM files and to sort alignments. Duplicate reads were identified and removed with the MarkDuplicates function of Picard tools v3.1.1. Read group information was added with AddOrReplaceReadGroups, and the BAM files were sorted with SortSam, both tools from the same suite. Base quality score recalibration (BQSR) was carried out with GATK’s BaseRecalibrator, incorporating known variant sites from Ensembl release 112 for *Bos taurus*.

**Fig. 2:**
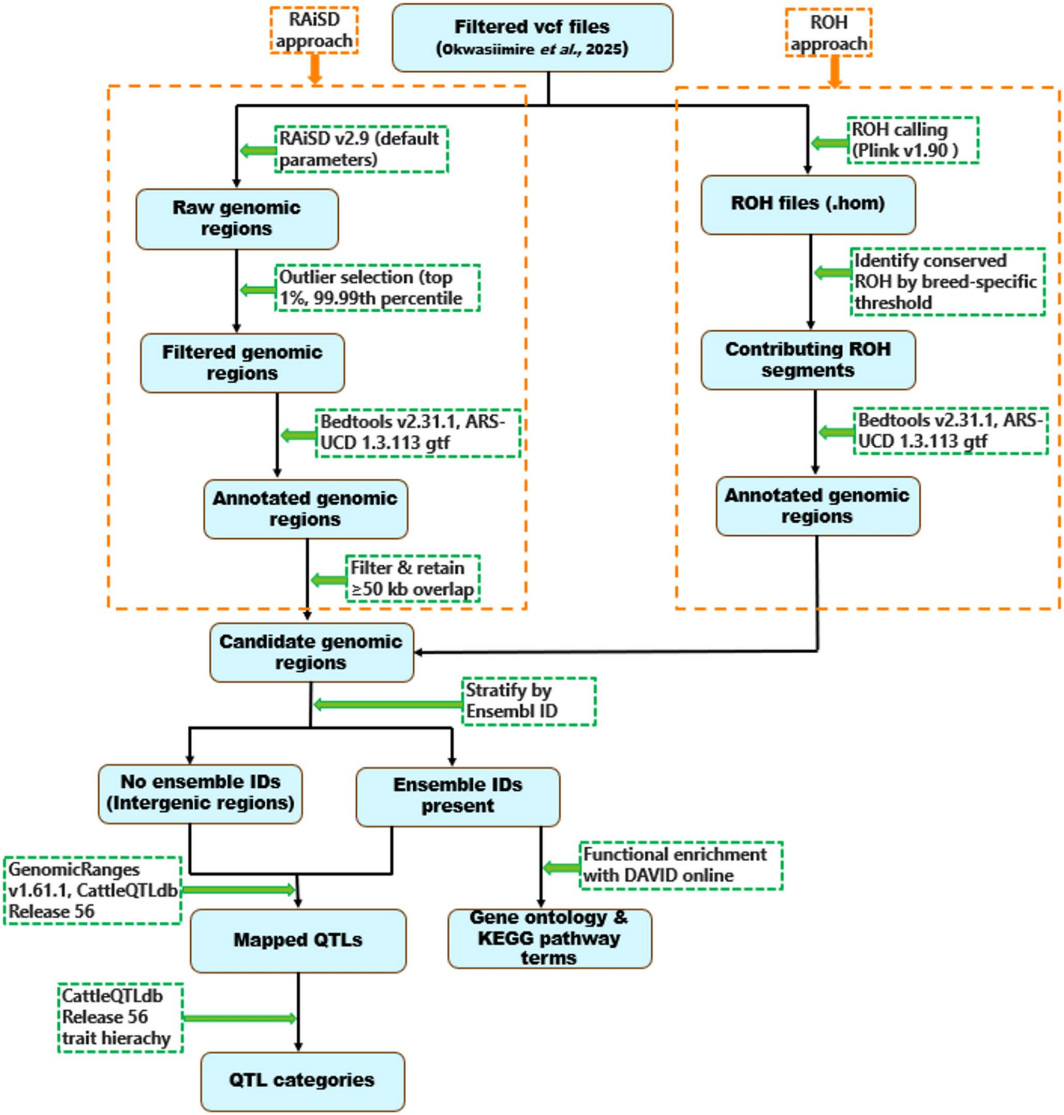
Steps followed for the identification of regions under putative selection with RAiSD and ROH analysis. Bioinformatics tools and actions are depicted by green arrows

Variants were discovered with GATK’s germline short variant workflow (Poplin et al. 2017). HaplotypeCaller in GVCF mode was used for variant calling per sample, and population GVCFs were merged with CombineGVCFs. Joint genotyping was performed with GenotypeGVCFs to obtain a consolidated VCF file. Joint genotyping ensures that all individuals are genotyped at the same genomic positions, producing a common set of SNPs across breeds. Filtering criteria to retain high-confidence variants was as follows: QD < 2.0, QUAL < 30.0, FS > 60.0, MQ < 40.0, SOR > 3.0, MQRankSum < − 12.5, and ReadPosRankSum < − 8.0. Only autosomal biallelic variants passing all quality filters and present in all individuals were retained for downstream analysis using GATK’s SelectVariants tool. Additional filtering of the variants was done to exclude those with call rates below 95% and minor allele frequencies (MAF) less than 0.05 using parameters -‐maf 0.05 ‐‐geno 0.05 in PLINK v1.90 (Chang et al. 2015). Furthermore, no individual samples were removed based on relatedness, and filters for minimum genotype call rate (0.8) and maximum missingness (0.2) were applied using VCFtools v0.1.17 (Danecek et al. 2011). The population substructure and genetic diversity assessment of these same samples has been comprehensively described by Okwasiimire et al. (2025). Two approaches (RAiSD and ROH) were then applied to the remaining jointly genotyped, high-confidence dataset of 22,455,225 variants to identify genomic regions under putative selection in the 95 animal genomes as shown in Fig. 2. Detailed descriptions of each method are provided in the subsequent sections.

### Identification and annotation of RAiSD-derived genomic regions

Potential signatures of selection were evaluated by calculating the µ-statistic as implemented in RAiSD v2.9 (Alachiotis and Pavlidis 2018) with default parameters. RAiSD calculates the µ-statistic, a composite measure that scores genomic regions while utilizing multiple signals of selection signatures including changes of the SFS, LD levels, and the genetic diversity along a given chromosome. Higher µ-statistic values indicate stronger evidence of positive selection. RAiSD analysis was performed separately for each of the six Ugandan cattle populations. To identify candidate genomic regions under selection, we first selected the top 1% of µ-statistic values on each autosome and then applied a stringent 99.99th-percentile cutoff within this subset (Pokharel et al. 2023). Briefly, the initial top 1% filter restricted the analysis to the strongest genomic signals, while the second percentile-based threshold isolated only the most extreme µ-statistic values. This two-step procedure minimized false positives and ensured that only the highest-confidence regions were retained as putative selective sweeps.

### Identification of runs of homozygosity

Runs of homozygosity (ROH) segments were identified for each of the six cattle populations using the --homozyg function in PLINK v1.90 (Chang et al. 2015). Briefly, the genome was scanned in sliding windows of 50 SNPs, allowing up to 2 heterozygous and 10 missing SNP calls per window. For each homozygous region, at least 2.5% of windows were required to be homozygous. Only ROH segments ≥ 500 kb in length, containing at least 50 SNPs, and with inter-SNP distances not exceeding 1000 kb at a minimum density of 1 SNP per 500 kb were retained.

Given that PLINK’s default ROH-calling parameters were originally optimized for lower-density SNP-array data, custom parameter settings were applied to ensure appropriate ROH segment detection in our short-read WGS data. Minimum ROH length thresholds were reduced (--homozyg-kb 500 vs. 1000; --homozyg-snp 50 vs. 100) to improve detection of shorter ROH and to retain segments in potentially SNP-sparse or unevenly covered regions. Other thresholds were increased (--homozyg-density 500 vs. 50; --homozyg-window-het 2 vs. 1; --homozyg-window-missing 10 vs. 5) to accommodate the genetic diversity and admixture status previously reported for Ugandan cattle populations (Okwasiimire et al. 2025), as well as potential reference bias arising from aligning *Bos indicus* genomes to the *Bos taurus*–derived ARS-UCD1.3 assembly.

To evaluate the robustness of our ROH detection parameters, we quantified concordance between F_ROH_ estimates obtained with default PLINK parameters for ROH detection and WGS-optimized parameters (for this study) at the individual level using Spearman and Pearson correlations. Similar parameter modifications have been implemented in recent WGS-based ROH studies such as Yasmin et al. (2023) and Minn et al. (2025), reflecting the need to adapt ROH-calling thresholds to dataset-specific properties.

### Detection and annotation of conserved runs of homozygosity

Both short and long homozygous segments can arise from mating among related individuals (Broman and Weber 1999); consequently, some observed homozygosity may reflect recent inbreeding, sampling from a limited gene pool, or selective pressures. In this study, all detected homozygous segments were considered regardless of length, as the primary goal was to investigate genes in conserved ROHs independent of inbreeding origin. The length of ROH classes can provide insights into the relative timing of inbreeding, with short ROH typically reflecting more ancient events and long ROH indicating more recent inbreeding (McQuillan et al. 2008; Purfield et al. 2012). We did not classify ROH by length in our analyses, as the main focus was on conserved regions across populations rather than the age of inbreeding events. For each cattle population,the inbreeding coefficient (F_ROH_) as derived from the detected runs of homozygosity by dividing the total length of ROH (L_ROH_) by the autosomal genome length (L_AUTO_) (McQuillan et al. 2008) was claculated. The autosomal genome length covered by the SNPs used in our analysis was 2,489,385.779 kb.

Conserved ROH segments were identified by calculating the frequency with which each SNP occurred in ROH segments across individuals in each population (Dixit et al. 2020; Ping et al. 2025). For each individual, SNPs located within at least one ROH segment were counted, and a per-SNP ROH-incidence value was obtained by summing the number of individuals in which that SNP occurred in a ROH. To define conserved ROH, a minimum-proportion threshold based on the number of genotyped individuals in each population was applied. Only ROH meeting the breed-specific threshold were considered for further analysis thus reducing the likelihood of including rare or individual-specific segments. SNPs with ROH-incidence values equal to or greater than the breed-specific cutoff were retained, and adjacent high-incidence SNPs were subsequently merged to delineate the underlying ROH regions. The ROH segments contributing to these high-incidence SNPs were then recovered by overlapping the selected SNP positions with the full set of breed-specific ROH intervals. For each breed, the number of contributing ROH segments and their genomic coordinates were extracted, and summary statistics were calculated for both the total ROH segments and those contributing to conserved regions. The identification of conserved ROH regions may be influenced by both sample size and the relatedness of sampled individuals. Although breed-specific thresholds were used to mitigate sampling bias, the composition of the sampled population can still affect the results. Therefore, conserved regions should be interpreted in the context of the populations analyzed.

Genome coordinates (chromosome number, start and end positions) of the identified genomic regions from RAiSD analysis as well as those for the ROH segments harboring common SNPs to each population were annotated with the *Bos taurus* genome assembly ARS-UCD1.3 gene transfer format (gtf) annotation file from Ensembl release 112 (Harrison et al. 2024) using BEDTools v2.31.1 (Quinlan and Hall 2010). For RAiSD data, only regions annotated with Ensembl gene ids, and which overlapped the positions in the annotation file by at least 50 kb (Koloi et al. 2025) were considered for subsequent analysis. Unique Ensembl gene identifiers were extracted from the retained regions of each cattle population and analyzed for intersections to identify those unique to each population as well as those shared with other breeds.

The Database for Annotation, Visualization, and Integrated Discovery (DAVID)Bioinformatics webserver (Huang et al. 2009; Sherman et al. 2022) was then used with default parameters for functional annotation, gene ontology, biological process and pathway enrichment analysis of the unique and shared genes. Only enrichment results with a Fisher’s exact *p-*value less than 0.05 (*p* < 0.05) were considered.

### Identification of quantitative trait loci from the cattle QTLdb

Cattle quantitative trait locus (QTL) and association data curated from published literature were downloaded from the Cattle QTL database (Cattle QTLdb), which is part of the Animal QTLdb (Release 56, April 24, 2025). The database facilitates the comparison, confirmation, and location of the most plausible location for genes responsible for quantitative traits useful to production in cattle (Hu et al. 2022).

The genome coordinates for regions unique to each cattle population (and those common to all breeds) were used to identify overlaps in the downloaded Cattle QTLdb data using the R-package GenomicRanges v1.61.1 (Lawrence et al. 2013). Due to overlap in trait names and QTL identifiers, we retained unique trait names from unique trait identifiers which were categorized following the cattle trait hierarchy implemented in the Cattle QTLdb. Following the hierarchy, all cattle trait names were grouped under six broad terms including health, meat and carcass, milk, production, reproduction, and exterior traits (Hu et al. 2022).

## Results

The average number of biallelic variants that passed all quality filters for each cattle population was as follows: Ankole (9,601,274 ± 209,593), Karamojong (11,255,505 ± 341,564), Nganda10 (9,918,257 ± 500,145), Nganda17 (10,106,759 ± 435,021), Nkedi (10,989,248 ± 403,203) and Ntuku (10,079,206 ± 291,976). Further details including the sequencing depth, mapping rate and coverage for individual samples are presented in Supplementary Table 1.

### Candidate genomic regions identified by RAiSD

RAiSD analysis identified 201,431 raw genomic regions as µ-statistic outliers across the six cattle populations. Filtering for regions overlapping the annotation file by at least 50 kb retained 895 genomic regions. A total of 803 candidate genes (those with Ensembl gene identifiers and associated gene names) were identified from the filtered regions and used for subsequent analysis.

Among the identified candidate genes were loci that lacked detailed functional annotation, including gene names or known biological roles in Ensembl release 112 (*Bos taurus* ARS-UCD1.3). These novel genes represent one of the three gene status classifications listed in Ensembl (others being “known” and “merged”) and correspond to coding sequences or loci with limited characterization in the primary databases underpinning Ensembl annotations including the European Nucleotide Archive (ENA), UniProtKB, NCBI RefSeq, The RNA families database (RFAM), miRBase, and tRNAscan-SE (Harrison et al. 2024). Such loci were classified as novel genes in this study. The Nganda10 population showed the highest proportion of filtered regions while the Nganda17 had the most retained candidate regions (1.1% and 93.1% respectively). The Ankole cattle had the highest number of novel regions (12.3%). Candidate regions with the highest mean µ-statistic values were observed in the Nganda10 animals (1.19 ± 0.676) (Table 1).

**Table 1:** Summary statistics of the identified genomic regions from RAiSD analysis of the six Ugandan cattle breeds

Breed	Raw genomic regions	Filtered genomic regions (%)	Novel (%)	Known		
				Count (%)	Mean µ_statistic	SD µ_statistic
Ankole	33,864	106 (0.3)	13 (12.3)	93 (87.7)	1.75E-11	1.33E-11
Karamojong	33,631	138 (0.4)	16 (11.6)	122 (88.4)	1.49E-11	1.07E-11
Nganda10	32,249	370 (1.1)	40 (10.8)	330 (89.2)	1.19E + 00	6.76E-01
Nganda17	33,876	101 (0.3)	7 (6.9)	94 (93.1)	1.94E-11	1.54E-11
Nkedi	33,907	91 (0.3)	9 (9.9)	82 (90.1)	1.92E-11	1.59E-11
Ntuku	33,904	89 (0.3)	7 (7.9)	82 (92.1)	1.87E-11	1.29E-11
Total	201,431	895 (0.4)	92 (10.3)	803 (89.7)		

Raw genomic regions = all the µ-statistic outliers, filtered genomic regions =  those retained after ≥ 50 kb overlap filtering, novel and known genes are derived from the filtered regions

**Fig. 3:**
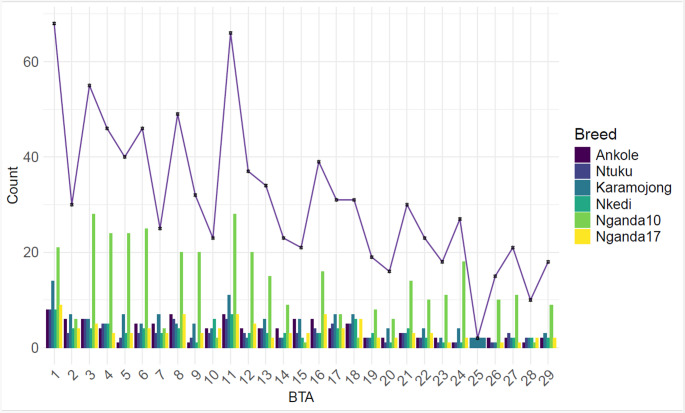
Distribution of the identified genomic regions from RAiSD analysis across the six Ugandan cattle populations. The trend line shows the number of genomic regions per chromosome (*Bos taurus* autosome, BTA) across all populations

Overall, the highest numbers of total filtered genomic regions were identified on BTA1 (*n* = 68), BTA11 (*n* = 66) and BTA3 (*n* = 55) while the lowest were on BTA25 (two regions) (Fig. 3, Supplementary Table 2: A and B). There was a strong positive correlation between chromosome length and the number of identified genomic regions. Specifically, chromosome length was positively correlated with the count of filtered candidate regions (*r* = 0.826, *p* = 0.000) and with the count of raw regions before filtering out the novel regions (*r* = 0.782, *p* = 0.000). This indicates that longer chromosomes tend to harbor more candidate regions, reflecting the effect of chromosome size on region detection.

### Annotation of RAiSD-derived genomic regions and QTLs

After excluding novel genes from the filtered regions per cattle population, intersection analysis was done to identify unique genes to each population and those common to all the breeds. A total of 39 common genes were found while breed-specific genes ranged from 330 for the Nganda10 animals to 82 each in the Nkedi and Ntuku animals (Table 3, Supplementary Table 2 A). The Ntuku and Nkedi cattle had candidate genes identified on 27 chromosomes, while the Ankole, Nganda10 and Nganda17 had candidate genes on 28 chromosomes. Only the Karamojong cattle had candidate genes identified on all the 29 chromosomes (Table 2, Supplementary Table 2 A).

**Table 2:** The annotated genes as identified by RAiSD on all 29 *Bos Taurus* autosomes (BTA) across the six cattle populations

BTA	Annotated genes*
1	MED12L, ZBTB38, HLCS, ANKRD28, HTR1F, ARL13B, HHLA2, IQCJ, SLC37A1, SH3BP5, APP, JAM2, FTL, CMSS1, IGSF11, GOLIM4, IQCJ, PLSCR1, NEK11, ADARB1, TNIK, NMD3
2	CMKLR2, ZBTB8A, ARHGEF10L, OSBPL6, R3HDM1, TANK, SCN1A, RAB3GAP1
3	MPZL1, IL6R, RAP1A, SLC6A17, CTH, LMX1A, ARHGEF11, GDAP2, CASQ2, COL11A1, BCAR3, RPAP2, GIPC2, ZZZ3, PATJ, DAB1, FYB2, FAM151A, LRP8, SCP2, AGBL4, IQCA1
4	CROT, ZNRF2, CHCHD3, LOC786254, VWC2, COL28A1, DGKB, MEOX2, SLC25A40, GRM3, SEMA3E, PLEKHA8, CPVL, COA1, ZNF800, SLC35B4, PTN, AGK, TPK1, UBE3C, PTPRN2, VIPR2
5	TUBA1C, GYS2, LOC101907335, ZDHHC17, NAV3, LIN7A, MGAT4C, SLC38A4, PTPRR, CPM, RASSF3, SCYL2, ITPR2, LMNTD1, BCAT1, AEBP2, PTPRO, SREBF2, SHISAL1, TBC1D22A, TAFA5
6	CFI, USP46, CD38, FAM193A, TECRL, MARCHF1, QRFPR, LOC104972658, CAMK2D, SLC9B1, ABCG2, KCNIP4, GABRG1, IGFBP7, MTHFD2L, ARHGAP24, NUDT9, JAKMIP1, C1QTNF7, ABLIM2
7	GATAD2A, LOC100299045, FGF1, SKIC3, TMEM232, SH3RF2, ZNF300
8	ELP3, ALDH1A1, PTCH1, MYTIL, FAM110C, CLCN3, SCARA5, PTPRD, PIP5K1B, VPS13A, GNA14, FRMPD1, SLC39A14, KIAA1958, CDK5RAP2, SH3YL1
9	LRP11, SERAC1, PACRG, LMBRD1, LOC132346195, CD109, MEI4, PKIB, AFG1L, FBXL4, GABRR2, TMEM200A, MOXD1, TIAM2, SLC22A3, PACRG, WDR27
10	ANP32A, TRAV10, TMOD3
11	LOC112448762, NCOA1, GRHL1, DDX31, FBLN7, NPAS2, MED27, MGAT4A, SLC4A5, TGFA, CRIM1, EML6, EDAR, EIF2AK3, UGP2, ARHGAP25, ANXA4, OTOF, DAB2IP, ABL1, AK8, NACC2, RXRA
12	HTR2A, TBC1D4, LOC107131273, DIAPH3, DNAJC15, SLC25A30, WDFY2, LHFPL6, DCLK1, SPATA13, TNFRSF19, SCEL, LOC100337390, ITGBL1, FGF14, MYO16, MCF2L
13	TASP1, RSU1, RIMS4, ZMYND8, SEL1L2, MALRD1, SIRPB1, PFKFB3, ARMC3, PTER, MPP7, SIRPB1, MAFB, TOX2
14	FER1L6, JPH1, TG, LYN, MTFR1, JPH1, CRISPLD1, BAALC, RGS22
15	DYNC2H1, LOC101903126, CAPN5, ALKBH8, PDHX
16	TPR, PROX1, LOC100336868, CFH, SPATA17, MARK1, COP1, TDRD5, HMCN1, KCNK2, LPGAT1, DENND1B
17	GUCY1B1, KNTC1, FSTL5, GLT1D1, SMAD1, RNF150, FBXW8, SEZ6L, WSCD2
18	CNGB1, CMTM4
19	RNF43, ANKFN1, EPN2, TANC2, ERN1, B3GNTL1, MAP2K6, SEPTIN9
20	GFM2, MRPS27, NDUFS4, CDH9, DNAH5
21	KLHL25, LOC101906604, GABRA5, AGBL1, LOC100298453, NUBPL, ARHGAP5, EGLN3, RIN3, PPP2R5C
22	FHIT, HRH1, FBXL2, SLC25A26, PTPRG, ERC2, HRH1, MRPS25
23	FARS2, DST, HMGCLL1, TINAG, CYP39A1, KIAA0319, ATXN1, ADTRP, OFCC1
24	MBD1, ALPK2, PIGN, CD226, CDH19, MAPRE2, GAREM1, COLEC12, ARHGAP28, PTPRM, RAB31, EPG5, KATNAL2, MAPK4, RAB27B
25	VWA3A, UBFD1
26	HTR7, TNKS2, EXOC6, AFAP1L2, TCERG1L
27	SH2D4A, NRG1, ZMAT4, SLC20A2, SH2D4A, PSD3, RARB, TOP2B
28	LRRC20, GNG4
29	LOC618367, PRCP, AAMDC, PAG20, SHANK2

*Some genes were annotated with pseudogene identifiers. See supplementary Table 2 A

A fraction of the candidate genes (20.7%; 166 of 803) was associated with functional enrichment results (*p* < 0.05) by the DAVID Bioinformatics online tool. These results included 33 Gene Ontology (GO) terms and 21 Kyoto Encyclopedia of Genes and Genomes (KEGG) pathways (Table 3, Supplementary Table 3: A and B). The KEGG pathways were related to signal transduction (thyroid hormone signaling pathway, Ras signaling pathway, cAMP signaling pathway, calcium signaling pathway, gap junction), immune function (pathways in cancer, inflammatory mediator regulation of TRP channels, complement and coagulation cascades, *Staphylococcus aureus* infection), cellular processes function (neuroactive ligand-receptor interaction) and lipid metabolism (lipid and atherosclerosis).

Majority of the enriched GO terms were exclusive or overlapping with Nganda10 cattle, with signal transduction (*n* = 18 genes) involving the highest number of genes. Other terms included brain development (*n* = 8 genes) in Nganda10 and Karamojong cattle, positive regulation of *ERK1* and *ERK2* cascade (*n* = 6 genes) enriched in Nganda10, Nganda17, Nkedi and Ntuku populations. Three GO terms including regulation of transcription by RNA polymerase II (*n* = 8 genes), positive regulation of transcription by RNA polymerase II (*n* = 4 genes), and positive regulation of endothelial cell migration (*n* = 2 genes) were enriched across all the 6 populations.

**Table 3:** Functional enrichment summary statistics of the unique and shared genes in each of the six Ugandan cattle breeds

Breed	Candidate genes	Functional enrichment		
		Associated genes	GO terms	KEGG pathways
Ankole	93	10	1	2
Karamojong	122	20	2	3
Nganda10	330	82	23	4
Nganda17	94	20	4	6
Nkedi	82	22	2	4
Ntuku	82	12	1	2
Common to the 6 breeds	39	10	3	1

The breed column depicts two categories: breed-specific (unique) and shared (common) genes to all the analyzed breeds

**Fig. 4:**
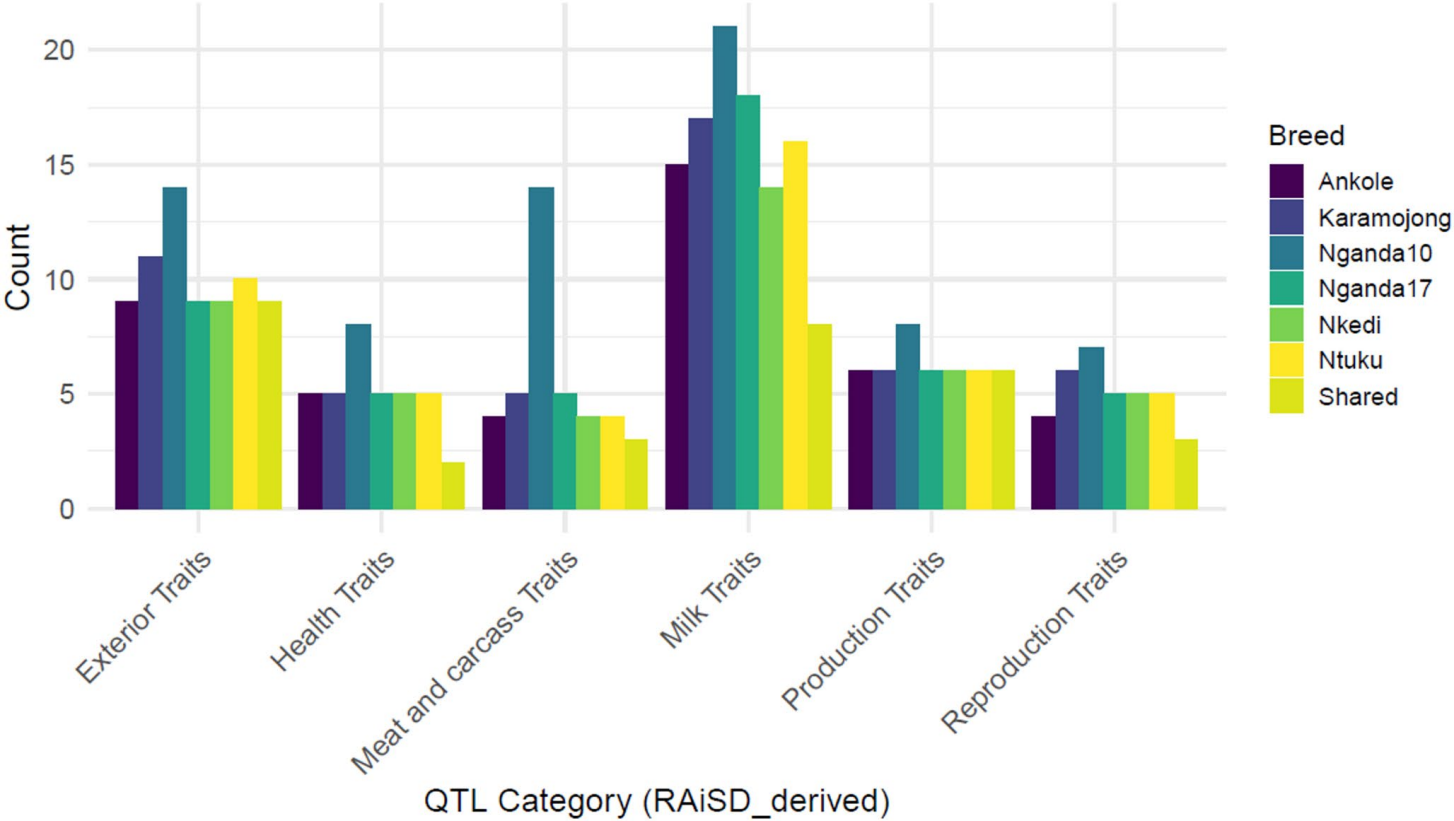
Distribution of the different traits (by trait hierarchy) identified by RAiSD from the unique genes in each of the six cattle populations

Autosome numbers and genome coordinates of the candidate genes were used to identify overlaps with quantitative trait loci contained in the Cattle QTLdb (Release56). For results that showed an overlap between QTL identifiers and QTL names, a single trait term was considered. In total, 302 unique QTL trait terms were retained across the 6 cattle populations ranging from 43 for Ankole and Nkedi cattle to 72 for Nganda10 cattle (Table 4, Supplementary Table 4: A and B). Overall, milk traits (33.4%) were the most prevalent, whereas reproduction traits (10.6%) were the least observed (Fig. 4).

**Table 4:** Summary of the quantitative trait loci identified from the RAiSD-derived candidate genes

Breed	Unique QTL names	Trait categories					
		Exterior	Health	Meat & carcass	Milk	Production	Reproduction
Ankole	43	9	5	4	15	6	4
Karamojong	50	11	5	5	17	6	6
Nganda10	72	14	8	14	21	8	7
Nganda17	48	9	5	5	18	6	5
Nkedi	43	9	5	4	14	6	5
Ntuku	46	10	5	4	16	6	5
**Shared**	**31**	**9**	**2**	**3**	**8**	**6**	**3**
Novel	16	0	2	1	2	0	1
Total (breed)*	302	62	33	36	101	38	32

The breed column depicts two categories: breed-specific (unique) and shared (common) genes as well as the novel genes identified across all breeds, *Total (breed) values exclude counts for Shared and Novel

### Genes and quantitative trait loci common to all the six breeds

The six cattle populations had 39 common genes located on 22 chromosomes, with BTA1 having the highest number (*n* = 6) (Supplementary Table 2). Only ten of the common genes returned functional enrichment results including three GO terms (regulation of transcription by RNA polymerase II, positive regulation of transcription by RNA polymerase II and positive regulation of endothelial cell migration) and one KEGG pathway (Thyroid hormone signaling pathway) (Table 3, Supplementary Table 3B). Searching the 39 genes in the CattleQTLdb returned 42 unique trait identifiers corresponding to 31 unique trait terms (Supplementary Table 4: A and B). Among these, exterior traits (*n* = 9) were the most frequent, while health-related traits (*n* = 2) were the least (Table 4; Fig. 4).

### Genomic regions identified from ROH-based methodology

The comparison of detected ROH with PLINK’s default settings and WGS-optimized parameters (for this study) showed high concordance, with individual-level F_ROH_ values exhibiting strong rank and linear agreement between the two parameterizations (Spearman *ρ* = 0.75; Pearson *r* = 0.77; both *p* < 10⁻¹⁸). In addition, the WGS-optimized parameters improved sensitivity to ROH detection, particularly for shorter ROH segments below 1 Mb (Online Resource 1). Given the higher marker density and uneven coverage characteristic of whole-genome sequencing data, we therefore adopted results derived using WGS-optimized ROH parameters for downstream analyses.

**Fig. 5:**
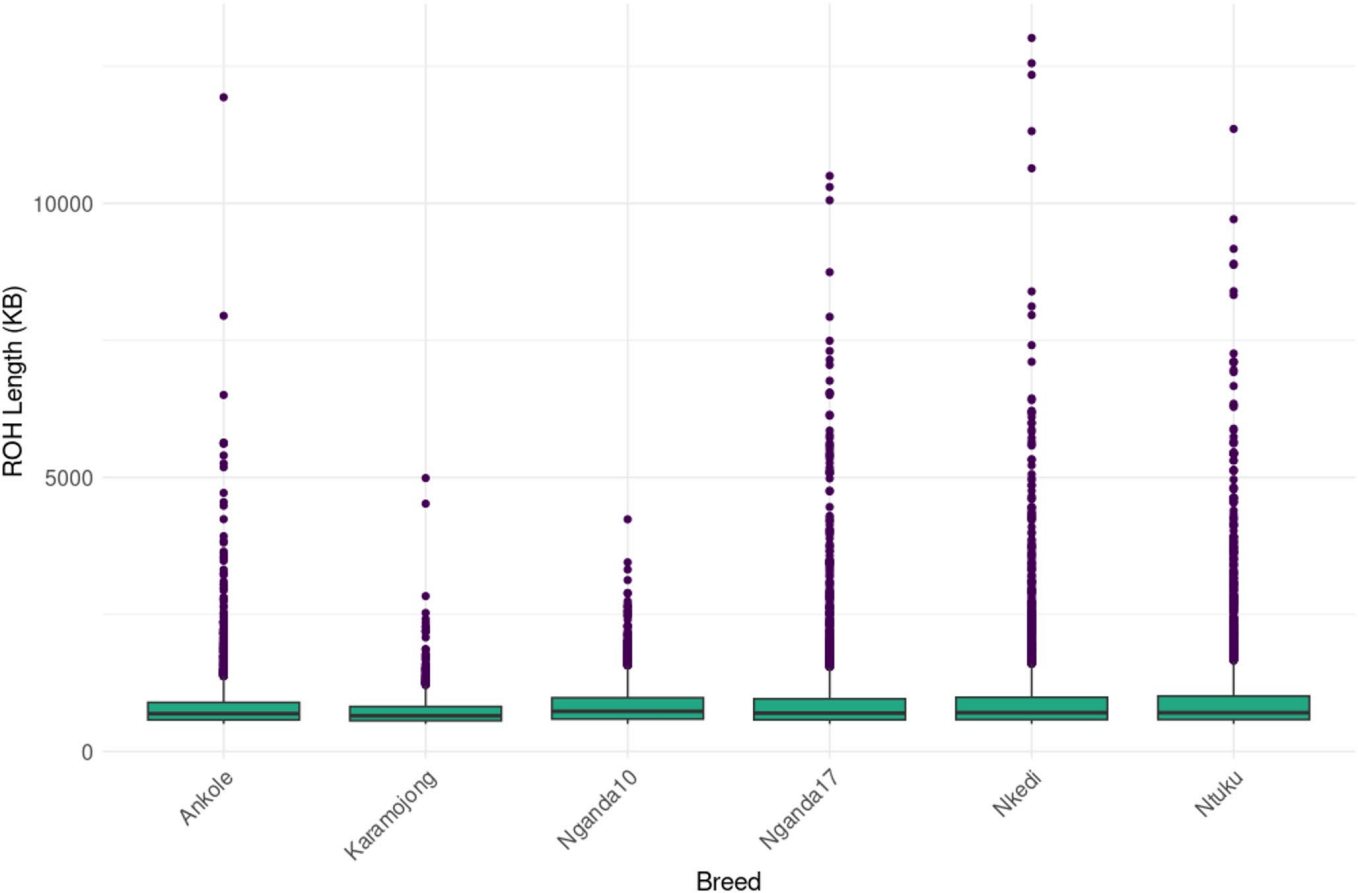
The distribution of ROH segments by size for each of the six cattle populations

**Fig. 6:**
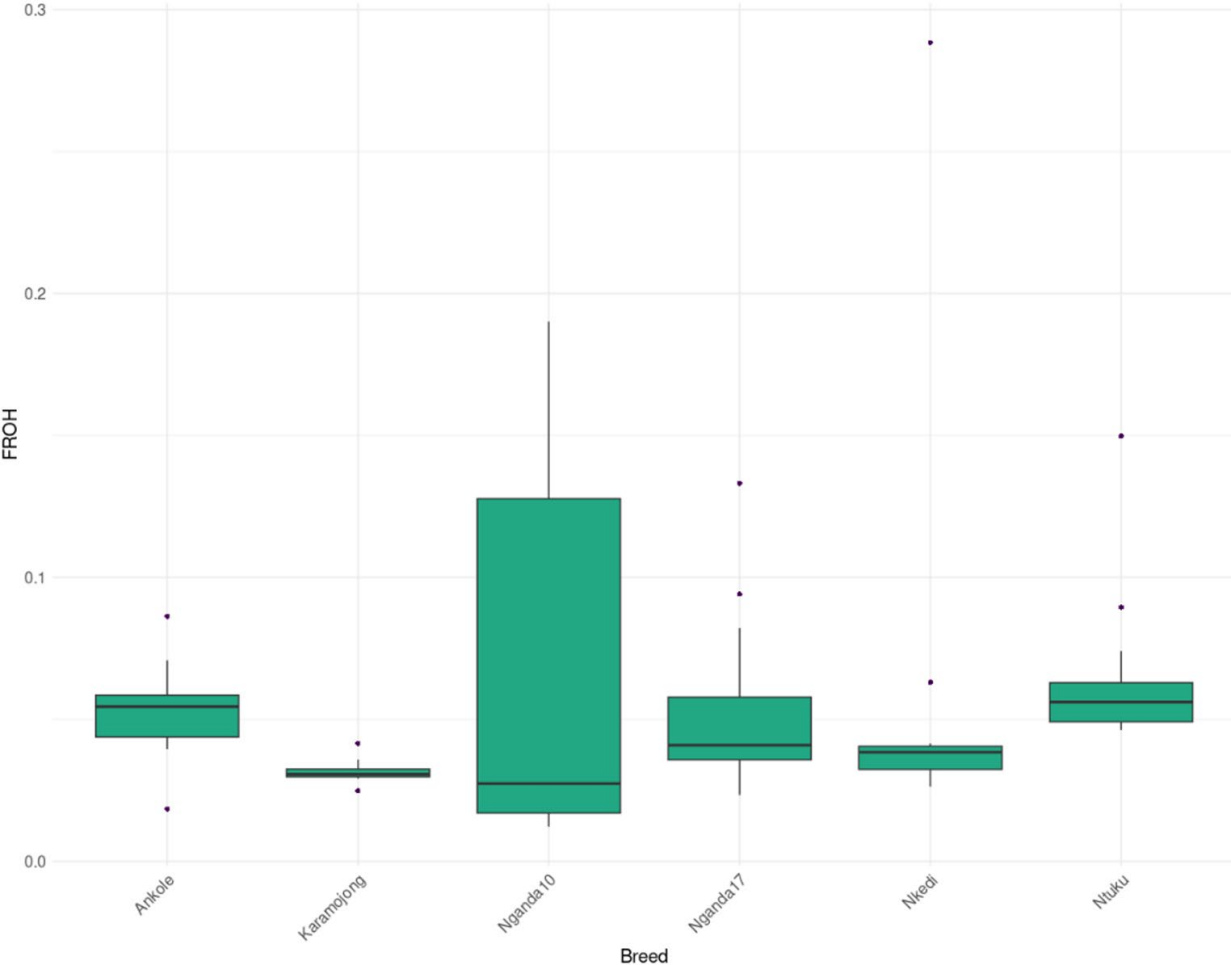
The distribution of the ROH-derived inbreeding coefficient (F_ROH_) in each of the six cattle populations

Across the six populations, 13,690 ROH segments were identified, with the highest number in Ntuku cattle (2,964 segments) and the lowest in the Karamojong cattle (1,166 segments) (Fig. 5, Supplementary Table 5 A). The number of sampled animals was strongly and positively correlated with the total number of ROH segments detected (*r* = 0.844, *p* = 0.035), indicating that larger sample sizes increased the likelihood of capturing more homozygous segments within a population. The size of segments observed per population (Fig. 5) and consequently the proportion of the genome in ROH (F_ROH_) varied across the different populations. The largest variation was observed for the Nganda10 while the least was seen in the Karamojong cattle (Fig. 6, Supplementary Table 5 A).

The proportion of animals required to identify shared SNPs per breed (contributing animals) ranged from 60% for the Ankole to 45% for Ntuku and Karamojong breeds (Supplementary Table 5 A). This pattern deviated from the expected trend in which the proportion of animals required to identify shared SNPs per breed would scale with sample size. Across populations, sample size showed a moderate negative but non-significant correlation with the number of conserved ROH (*r* = − 0.662, *p* = 0.152), indicating that the detection of conserved ROH segments was not primarily driven by the number of sampled animals. For example, the Nganda10 (*n* = 10) and Nganda17 (*n* = 17) groups both exhibited common segments in 50% of the animals, whereas the Karamojong (*n* = 11) and Ntuku (*n* = 19) breeds showed shared runs in 45%. In contrast, the Ankole and Nkedi (both with *n* = 19) displayed shared segments in 60% and 55% of individuals, respectively. In addition, there was no observed relationship between the number of conserved ROH segments and the number of contributing animals (*r* = 0.0, *p* = 1.000). Collectively, these results suggest that other factors beyond sample size, such as breed-specific levels of genetic diversity or differences in selection pressure contribute to the likelihood of detecting common homozygous segments.

Likewise, the number of shared SNPs ranged from 12,013 SNPS (in 31 ROH segments) in the Nganda10 animals to 675 SNPs (in 11 ROH segments) in Nkedi cattle (Supplementary Table 5B). A total of 108 ROH segments (contributing segments) with 23,967 shared SNPs were identified across the six cattle populations. Nganda10 cattle had contributing segments on five chromosomes (BTAs 6, 9, 11, 20 and 24), the highest being on BTA24 while the Ankole and Nkedi animals had segments on one chromosome apiece (BTAs 5 and 7, respectively) (Supplementary Table 5B). The 108 identified segments were then annotated with the *Bos taurus* genome assembly ARS-UCD1.3 gtf annotation file returning 49 candidate genes in total (Tables 5 and 6, Supplementary Table 6). Nganda10 cattle had the highest number of candidate genes (*n* = 14) while Ankole animals had only three genes. Notably, functional enrichment (*p* < 0.05) of candidate genes from the six cattle populations using the DAVID Bioinformatics online was successful only for Ntuku cattle, returning only two GO terms: protein metabolic process (GO:0019538) and macromolecule metabolic process (GO:0043170).

**Table 5:** Summary of the runs of homozygosity analysis

Breed	All segments					Conserved segments				
	No. animals	No. ROH	SNPs in ROH (%)	F_ROH_		Breed-specific threshold	Shared ROH	Candidate genes identified	Mean size (kb)	Novel (%)
				Mean	SD					
Ankole	19	2938	6,028,475 (51.1)	0.052	0.014	0.6	12	3	1732.19 (± 726.6)	1 (33.3)
Karamojong	11	1166	3,716,376 (24.9)	0.032	0.004	0.45	16	7	1092.82 (± 766.55)	1 (14.3)
Nganda10	10	1950	6,070,609 (44.8)	0.067	0.069	0.5	31	14	2138.62 (± 2837.64)	5 (35.7)
Nganda17	17	2232	6,570,952 (51.1)	0.051	0.028	0.5	18	7	969.06 (± 357.18)	2 (28.6)
Nkedi	19	2440	7,542,463 (56.7)	0.051	0.058	0.55	11	8	943.23 (± 1069.77)	3 (37.5)
Ntuku	19	2964	7,338,371 (62.2)	0.062	0.024	0.45	20	10	837.21 (± 268.75)	3 (30.0)
Total	95	13,690	37,267,246 (47.2)				108	49		15 (30.6)

**Table 6:** The annotated genes from the contributing ROH segments *Bos Taurus* autosome (BTA) location across the six cattle populations

BTA	Annotated genes
5	IL26, GRIP1
7	OR2AV11, C2CD4C, EFNA2, PWWP3A, WNT8A, NME5, GFRA3, CTH, PAIP2, MYOT, CDC23, ETF1, HSPA9
9	A0AAA9TAP0_BOVIN, A0AAA9RVN8_BOVIN
11	NT5C1B, KCNS3
12	RFXAP, SERTM1, SOHLH2
16	RERE
20	FBXL7, ANKH
23	KHDRBS2
24	RBM17, NOL4, ASXL3, CCDC178

### Quantitative trait loci identified from ROH-derived genomic regions

Using genomic coordinates, the Cattle QTLdb (Release 56) was queried for quantitative traits associated with the selected ROH segments from each of the six cattle populations. The analysis identified 1,138 trait terms corresponding to 696 QTL identifiers (Supplementary Table 7 A). After filtering to remove repeated identifiers and terms, 223 unique QTLs were retained. Across all populations, milk-related traits constituted the majority, except in Ankole cattle, where meat and carcass traits (*n* = 11) were predominant (Table 7, Supplementary Table 7B). This observation aligns with previous genomic evidence indicating that Ankole cattle possess strong selection potential for superior meat quality traits (Taye et al. 2017b).

**Table 7:** Quantitative trait loci identified from the ROH-derived candidate genes

Breed	BTA (*n*)	Mapped regions	Raw QTLs	Unique QTL Ids	Unique QTL terms	Trait categories					
						Exterior	Health	Meat & carcass	Milk	Production	Reproduction
Ankole	1	3	324	179	35	3	4	11	5	4	8
Karamojong	3	7	101	53	29	6	2	5	7	4	5
Nganda10	5	14	358	283	80	12	7	9	37	8	7
Nganda17	2	7	73	37	25	3	0	7	7	3	5
Nkedi	1	8	112	25	13	0	1	4	5	3	0
Ntuku	2	10	170	119	41	8	1	7	12	3	10
Novel	8	15	**1009**	**951**	**247**	**41**	**20**	**46**	**62**	**35**	**43 **
Total (breed)*	-	-	1138	696	223	32	15	43	73	25	35

Raw QTLs = all the mapped QTLs, Unique QTL Ids = exclude repeated QTL identifiers, Unique QTL names = exclude repeated QTL terms, the breed column depicts two QTL categories: those identified from breed-specific (unique) and those derived from the novel regions identified across all breeds, *Total (breed) values exclude counts for Novel

### Genomic regions classified as novel genes

Following the annotation of candidate genomic regions with the *Bos taurus* genome assembly ARS-UCD1.3 gtf file, some regions were found to contain Ensembl gene identifiers but lacked associated gene names or pseudo-gene annotations. These regions were designated as novel genes, defined as transcripts that do not match any sequences in established public scientific databases such as ENA, UniProtKB, NCBI RefSeq, RFAM, miRBase, and tRNAscan-SE (Harrison et al. 2024). Novel genes are thought to originate from non-coding genomic regions that subsequently gain functions, leading to their preservation through natural selection. Such regions have been observed to be under selection in mammals, including humans and chimpanzees (Ruiz-Orera et al. 2015). The novel genes identified in this study may be considered species-specific to *Bos taurus*, as homolog searches were limited to the Ensembl database, which designates novel genes using a species-specific approach. No significant correlation was observed between chromosome length and the number of novel candidate regions (*r* = 0.153, *p* = 0.427) detected by RAiSD. A weak positive correlation was observed between chromosome length and the total number of detected regions before filtering (*r* = 0.321, *p* = 0.089), suggesting that while chromosome size influences the overall number of regions identified, it does not strongly affect the distribution of novel regions.

**Fig. 7:**
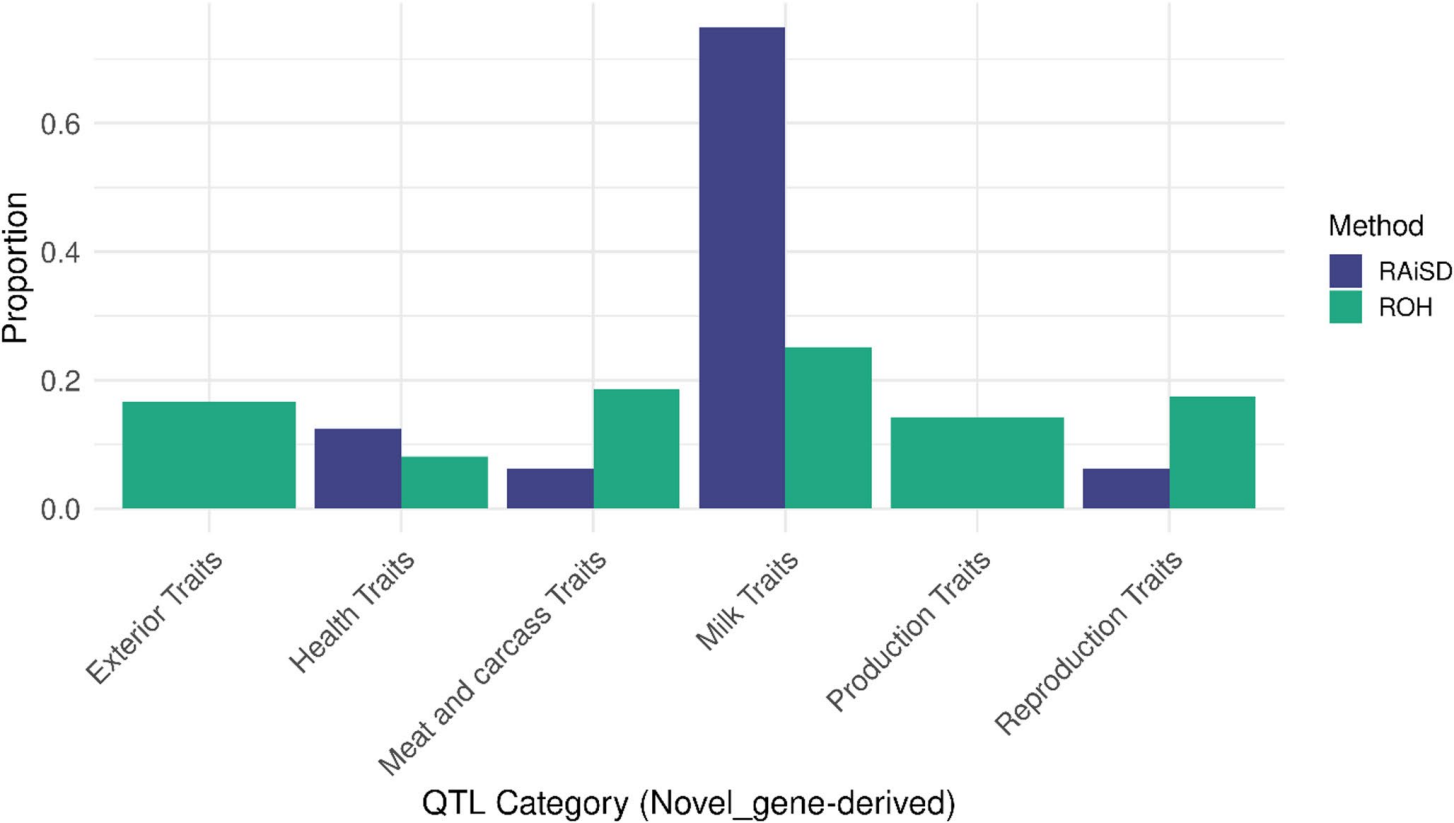
Proportions of the different traits (by trait hierarchy) identified from genome coordinates of novel genes derived from both RAiSD and ROH across the six cattle populations

In total, RAiSD identified 92 novel genes (10.3%) (Table 1, Supplementary Table 8 A) while ROH analysis detected 15 novel genes (30.6%) (Table 5, Supplementary Table 9). RAiSD-derived novel genes overlapped with eight QTL terms (Table 4, Supplementary Table 8B), whereas ROH-derived novel genes were linked to 247 terms spanning all trait categories (Table 7, Supplementary Table 9). Comparison of QTL associations between RAiSD and ROH (Fig. 7) revealed fewer terms from RAiSD-derived novel regions (92 genes, 16 terms; Supplementary Table 8B) compared to ROH-derived terms (15 genes, 247 terms; Supplementary Table 9). Whereas novel genes may give rise to RNA genes and, in some instances, protein-coding genes (Schmitz and Bornberg-Bauer 2017), a BLAST search in the Ensembl database on 15 randomly selected novel genes detected in this study indicated that they are predominantly long non-coding RNAs (lncRNAs). This finding underscores the need for continued efforts to improve annotation of the cattle genome.

## Discussion

This study reports novel insights into the genomic architecture of Ugandan indigenous cattle populations, highlighting candidate regions under putative selection, including *Bos taurus*-specific novel genes. Several of the identified regions overlap with economically important QTLs, associated with adaptation to tropical environments, disease resistance, and production. This work expands on previous studies on native cattle across Africa, uncovering previously uncharacterized genomic regions. Identification of such regions is particularly relevant for preserving the genetic diversity of native cattle which might otherwise be lost through introgression with commercial breeds or breed replacement, thereby maintaining variation associated with key adaptive and productive traits in African cattle.

### Genomic regions detected by RAiSD and ROH analysis

RAiSD analysis identified 895 candidate genomic regions encoding 803 genes, including 92 novel genes. ROH analysis detected a total of 108 segments across the six cattle populations. The segments harbored 49 genes of which 15 were novel. Notably, neither the genic nor the novel regions identified by the two methods shared gene identifiers or names. This lack of overlap likely reflects the different biological patterns captured by each method: RAiSD detects changes in the site frequency spectrum, levels of linkage disequilibrium, and local genetic diversity (Alachiotis and Pavlidis 2018), whereas ROH represent conserved genomic segments with limited variation (Vitti et al. 2013). The absence of jointly identified putative selection signatures in our study contrasts with results in Red Angus, where 12 sweep regions were detected by both RAiSD and the haplotype-based statistic nSL (Rowan et al. 2024). However, our findings are not exceptional, as several studies have reported minimal or no overlap among methods applied to the same dataset. For instance, in chickens, ROH analyses and three EHH-based approaches did not identify any shared candidate regions (Mastrangelo et al. 2023). In Valdostana cattle, only two regions were jointly detected by *F*_*ST*_, EHH-based statistics (*iHS* and *Rsb*), and ROH (Mastrangelo et al. 2020). Likewise, in Hanwoo and Angus cattle, only 83 SNPs were shared between ROH and *Rsb* (Ju et al., 2025). These examples illustrate that limited concordance among selection-scan methods is common and reflects their sensitivity to distinct evolutionary signals.

ROH analysis revealed a positive correlation between sample size and the total number of detected ROH segments across populations, consistent with the expectation that larger datasets capture greater within-population genomic diversity and a wider spectrum of homozygosity. In contrast, the number of conserved ROH (regions shared by multiple individuals in a population) did not show significant correlation with sample size. The observed perfect correlation between the number of conserved ROH and the number of individuals in which they were observed (contributing animals) was expected, as conserved ROH are inherently defined based on shared homozygosity across animals. These patterns indicate that while sample size influences the total ROH segments detected, it does not substantially bias the identification of conserved ROH at the population level. Consequently, the conserved ROH reported in this study likely reflect genuine population-level genomic features rather than artifacts of sampling.

Outstandingly, the Nganda10 population displayed exceptionally high mean µ-statistic values (1.19), far exceeding those of all other breeds, which exhibited mean values close to zero. This strong signal coincided with the highest mean F_ROH_ (0.067 ± 0.069), whereas the remaining populations showed lower and more uniform homozygosity levels. Together, the elevated µ-statistic and increased F_ROH_ indicate that Nganda10 exhibits genomic patterns distinct from the other breeds. As reported by Okwasiimire et al. (2025), the Nganda10 population evolved from a nucleus herd that was established as a conservation population. Such populations are characterized by closed and isolated breeding structures with restricted mating patterns, limited gene flow and reduced heterozygosity. These demographic characteristics could have amplified the composite µ-statistic by increasing the individual µ components (µ_VAR, µ_SFS, and µ_LD) (Alachiotis and Pavlidis 2018).

### Association of genes in regions under putative selection with traits of importance in cattle

The genes annotated from genomic regions identified as being under putative selection in the Ugandan indigenous cattle were categorized based on economically important traits (Table 8). This study provides detailed discussion of categories related to parasite, vector, disease, and infection-related traits; adaptation to the tropical environment; and traits associated with temperament and farmer preferences. Although genes associated with production, reproduction, and growth traits were also identified within the candidate regions, these were not the primary focus of this study. Indigenous cattle in Uganda are kept mainly for their resilience, adaptation to harsh conditions, and multifunctional use, and our analyses were therefore oriented toward genes underlying these traits. Nonetheless, several genes related to production and reproductive performance were present in the detected regions, indicating that natural and low-intensity artificial selection may also influence such economically important traits in these populations. A summary of these genes is included in Table 8 to provide a complete representation of all the detected functional categories.

**Table 8:** Stratification of the genes under putative selection by trait category

Trait category	Genes	Method
Parasite, vector, disease and infection related*	MED12L, IL6R, TBC1D4, A0AAA9SLV6_BOVIN (ENSBTAG00000059884), CHCHD3, LOC107131273, RSU1, TIAM2, TECRL, SEZ6L, AGBL1, LRP8, SCN1A, PTPRO, CPVL, KLHL25	RAiSD
	IL26, OR2AV11, FBXL7, POSTN, HSPA9	ROH
Adaptation to the tropical environment*	SHANK2, PTPRD, CPVL, ITPR2, RAB31, EIF2AK3, A0A3Q1N3Q4_BOVIN (ENSBTAG00000058910), SLC37A1, AGBL1, COA1, GABRG1, KHDRBS2	RAiSD
	GRIP1, HSPA9	ROH
Farmer preferences and temperament*	A0AAA9SS40_BOVIN (ENSBTAG00000061529), OSBPL6, ZZZ3, LMNTD1, PTPRO, GABRG1, ANKFN1, FBXL2, USP46, RAB3GAP1, SPATA17, SHANK2	RAiSD
	KHDRBS2, FBXL7, ASXL3, CCDC178	ROH
Production related**	MED12L, HLCS, A0AAA9SLV6_BOVIN (ENSBTAG00000059884), A0A3Q1N3Q4_BOVIN (ENSBTAG00000058910), A0AAA9SS40_BOVIN (ENSBTAG00000061529), DDX31, TBC1D4, RSU1, SH2D4A, GNG4, OSBPL6, IL6R, KIAA0319, PSD3, SMAD1, SPATA17, NEK11, SCN1A, ZZZ3, SLC38A4, COA1, LMNTD1, PTPRO, ZNF300, CNGB1, TECRL, SLC37A1, ABCG2, GABRG1	RAiSD
	KHDRBS2, NOL4, POSTN, SOHLH2, MYOT	ROH
Reproduction and growth-related**	HLCS, IL6R, CHCHD3, RSU1, SH2D4A, A0A3Q1N3Q4_BOVIN (ENSBTAG00000058910), FBXL4, CPVL, TIAM2, CNGB1, TECRL, OSBPL6, PTPRO, RAB3GAP1, VWA3A, SMAD1, TAFA5, LRP8, SCN1A, AGBL1, SLC38A4, COA1, GABRG1, LMNTD1	RAiSD
	IL26, KHDRBS2, NT5C1B, FBXL7, POSTN, WNT8A	ROH

RAiSD = Raised Accuracy in Sweep Detection, ROH = runs of homozygosity analysis, * = presented in the discussion section ** = not discussed in this study

### Traits related to parasites, vectors, diseases and infections

Traits related to animal health are of significant value in cattle genetics due to their profound impact on the productivity and economic returns of animal enterprises. In the tropics, animals are continuously exposed to parasites, vectors and the diseases they transmit, including protozoan and helminth infections, as well as bacterial and viral diseases (Byaruhanga et al. 2017). Vectors such as ticks, tsetse flies and nuisance flies exacerbate the stress placed on livestock. The ability of animals to resist or tolerate these stress factors while continuing to produce valuable commodities like milk, meat and draught power to their owners potentially shaped historical breeding decisions in favor of better adapted animals. Overtime, the practices of cattle keepers left detectable patterns on the genomes of their animals in form of signatures of selection.

Several genes with known associations to bovine health-related traits were shared across all six Ugandan cattle populations. Among these, *MED12L* (*mediator complex subunit 12 L;* BTA1) and *IL6R* (*interleukin 6 receptor;* BTA3) have been associated with mastitis in cattle (Wang et al. 2022; Zhang et al. 2018). In addition, *TBC1D4* (*TBC1 domain family member 4;* BTA12), previously linked to health traits in American Holstein (Cole et al. 2011), and the pseudogene *A0AAA9SLV6_BOVIN* (ENSBTAG00000059884; *Endonuclease/exonuclease/phosphatase domain-containing protein;* BTA6), which maps to QTLs 179659 and 122681 associated with health and production traits in Holstein (Nayeri et al. 2019) were also identified. Other notable genes included *CHCHD3* (*coiled-coil-helix-coiled-coil-helix domain containing 3;* BTA4) which has been associated with susceptibility to bovine coronavirus and bovine respiratory disease infections in American cattle (Kiser and Neibergs 2021), and *LOC107131273* (*ATP-binding cassette sub-family C member 4;* BTA12), that maps to QTL 288172 related to health traits in lactating Holstein (Siberski-Cooper et al. 2024). These two genes were located within candidate regions under putative selection in all populations except Nganda10. The *LOC107131273* gene has previously been implicated in *Escherichia coli* induced clinical mastitis in Holstein (Cheng et al. 2022), and large offspring syndrome in Angus crossbred heifers (Rivera et al. 2022). Similarly, *RSU1* (*Ras suppressor protein 1;* BTA13), a candidate gene for mastitis resistance in Italian Holstein (Strillacci et al. 2023), was detected in all populations except Nganda10 and Karamojong. The identification of several mastitis-associated genes across Ugandan indigenous cattle highlights their potential role in conferring resistance to mastitis, a disease of major economic and animal welfare concern in Uganda’s dairy production systems (Byaruhanga et al. 2017; Kakooza et al. 2023).

In addition to the genes common to all cattle populations, distinct population-specific genes were identified. *TIAM2* (*TIAM Rac1 associated GEF 2;* BTA9, Ntuku and Nganda10) has been previously associated with mastitis in Holstein (Siebert et al. 2018), and with hepatic fibrinogen storage disease in Wagyu cattle (Jacinto et al. 2023). *TECRL* (*trans-2*,*3-enoyl-CoA reductase like;* BTA6, Nganda17 and Karamojong) has been linked to bovine respiratory disease and lung consolidation in Holstein calves (Quick et al. 2020). *SEZ6L* (*seizure related 6 homolog like;* BTA17, Nganda10), has been associated with resistance to foot-and-mouth disease (Lee et al. 2014). Similarly, *IL26* (*interleukin 26;* BTA5, Ankole), has been linked to productive performance and health status of transition dairy cattle (Zhang et al. 2019), and has been suggested as a potential marker in breeding for resistance to *S. aureus* induced mastitis (Wang et al. 2020) and *Mycoplasma bovis* infection in cattle (Correia et al. 2022). Other genes included *AGBL1* (*AGBL carboxypeptidase 1;* BTA21, Nganda10), which has been linked to leukocyte telomere length in cattle (Igoshin et al. 2023), and *POSTN* (*periostin;* BTA12, Nganda17), that has been reported as a candidate marker for subclinical endometritis (Tobolski et al. 2024). Similarly, *LRP8* (*LDL receptor related protein 8;* BTA3, Nganda10) has been associated with immune system function in Nelore cattle (Dos Santos et al. 2025). In Karamojong cattle, *OR2AV11* (*olfactory receptor family 2 subfamily AV member 11;* BTA7), belonging to the olfactory receptor genes responsible for olfaction (Lee et al. 2013), was identified. In Nganda10, several other genes were detected, including *SCN1A* (*sodium voltage-gated channel alpha subunit 1;* BTA2) studied in relation to trypanotolerance in West African cattle (Goyache et al. 2021) and *PTPRO* (*protein tyrosine phosphatase receptor type O;* BTA5) associated with *M. bovis* and bovine viral diarrhea virus infection (Goldkamp et al. 2025). The *FBXL7* (*F-box and leucine rich repeat protein 7;* BTA20) gene, which has been linked to clinical mastitis and clinical ketosis in dairy cattle (Nayeri et al. 2019) as well as lipomatous myopathy in Piedmontese cattle (Peletto et al. 2017) was also detected in Nganda10.

With respect to tick resistance, *LOC107131273* mapped to QTL 288172 (mean corpuscular hemoglobin concentration, MCHC). This gene was also identified in a study on resistance to tropical theileriosis in Portuguese cattle (Valente et al., 2024)

The genes *CHCHD3*, *IL26* (Ankole) as well as *CPVL* (*carboxypeptidase vitellogenic like;* BTA4, Nganda10) have been reported as putative signatures of resistance to *Rhipicephalus microplus* tick infestation (Carvalho et al. 2024; Moré et al. 2019). Similarly, *HSPA9* (*heat shock protein family A [Hsp70] member 9;* BTA7, Ntuku) has been associated with trypanotolerance in Sheko cattle (Mekonnen et al. 2019), while *KLHL25 (kelch like family member 25;* BTA21, detected in all populations except Nganda10) has been reported as a candidate gene for resistance to vector borne infections in African indigenous cattle (Kambal et al. 2023). Furthermore, QTL 288172, associated with MCHC, was present across all six cattle populations. MCHC is a red blood cell index commonly used alongside other hematological indices to assess anemia and red blood cell morphology. Both conditions are key diagnostic indicators of several tropical animal diseases including trypanosomosis, tick-burden and tick-borne infections such as theileriosis, babesiosis and anaplasmosis (Roland et al. 2014; Turkson and Ganyo 2015).

Collectively, these findings demonstrate the genomic basis of cattle adaption to the parasite and vector-laden Ugandan ecosystem, where ticks, tsetse flies and vector-borne infections pose persistent challenges (Byamukama et al. 2021; Byaruhanga et al. 2021; Etiang et al. 2024; Kizza et al. 2021; Mandela et al. 2020). They also underscore the potential of Ugandan indigenous cattle to serve as valuable reservoirs of genetic variation for enhancing disease resistance and resilience in African production systems.

### Traits related to adaptation to the tropical environment

The majority of indigenous animals in Uganda are reared under traditional management systems, characterized by open grazing on communal lands, pastoral rangelands or tethering in agropastoral areas (Kabi et al. 2016; Kugonza et al. 2011). Livestock maintained in such low input systems are constantly exposed to high stress arising from constant migration, drought, feed and water shortages, parasites, vectors and vector-borne diseases. As adaptative strategies, indigenous cattle have developed several unique characteristics that are pertinent to their fitness. These features are related to effective feed and water utilization, energy metabolism and conservation, withstanding elevated temperatures and exposure to vectors and associated diseases (Rege et al. 2011; Wilson 2009).

In this study, the KEGG pathway term bta04919 (Thyroid hormone signaling pathway) was found enriched across all the 6 cattle populations. Thyroid hormones, secreted by the thyroid gland, play a key role in regulating growth, development and metabolism (Bianco et al. 2019). As previously noted, native cattle are typically managed under low input systems; thus, this pathway may be under selection as an adaptation mechanism to reduce energy expenditure during periods of reduced feeding and restriction of calories (De Andrade et al. 2015). Such calorie deficits are common during dry spells and prolonged droughts, when feeds and water are scarce; scenarios frequently experienced in tropical environments such as Uganda’s cattle corridor (Nalwanga et al. 2024).

In the Nganda10 population, several genes associated with heat and environmental adaptation were identified. *SHANK2* (*SH3 and multiple ankyrin repeat domains 2;* BTA29) has been reported in relation to heat tolerance (Wang et al. 2017). *PTPRD* (*protein tyrosine phosphatase receptor type D*; BTA8) plays a regulatory role in drinking behavior, and has been identified under selection for drought and climatic adaptation (Cai et al. 2025; Porto-Neto et al. 2014). The *CPVL* gene has been implicated in desert environment adaptation of sheep (Yang et al. 2016), while *ITPR2* (*inositol 1*,*4*,*5-trisphosphate receptor type 2*; BTA5) has been linked to thermotolerance in African cattle (Taye et al. 2017a), environmental adaptation (Terefe et al. 2023) and high altitude adaptation in Ethiopian cattle (Terefe et al. 2022).

*RAB31* (*RAB31*,* member RAS oncogene family*; BTA24), enriched for GO:0006897 (endocytosis), was detected under selection in Nganda10 and has been linked to coat color and consequently heat tolerance in Dehong humped cattle (Li et al. 2020). *AGBL1* has been studied in relation to differentiation of adipocytes (Zhou et al. 2025), while *COA1 (cytochrome c oxidase assembly factor 1;* BTA4) has been identified as a candidate gene for heat stress resistance in Brown Swiss cattle (Nuñez Soto et al. 2023). Moreover, *GABRG1 (gamma-aminobutyric acid type A receptor subunit gamma1;* BTA6) has been associated with adaptation to the tropical African environment in Nigerian cattle (Mauki et al. 2022).

In other cattle populations, the genes *HSPA9* (detected in Ntuku) and *GRIP1* (*glutamate receptor interacting protein 1*; BTA5, detected in Ankole), have been linked to tropical adaptation in Ethiopian Abigar cattle (Ayalew et al. 2023). In the Karamojong cattle, the GO term GO:0006974 (DNA damage response) was enriched, potentially reflecting adaptation to heat stress. Heat stress has previously been shown to cause oxidative stress and DNA damage (Houston et al. 2018). In this regard, *EIF2AK3* (*eukaryotic translation initiation factor 2 alpha kinase 3;* BTA11, Nganda10), which belongs to EIF2alpha kinases that are activated by oxidative stress, viral infections, nutrient deprivation and endoplasmic reticulum stress (Carter 2007) was found under selection and enriched for the KEGG pathway term bta05417 (Lipid and atherosclerosis). A related gene, *EIF2AK4*, which is also involved in oxidative stress and DNA damage repair, has been associated with heat tolerance in Chinese cattle (Wang et al. 2019).

Other genes include *A0A3Q1N3Q4_BOVIN* (ENSBTAG00000058910; BTA18, detected in all breeds except Nganda10), reported as a potential target in breeding for environmental adaptability in South African crossbred cattle (Kooverjee et al. 2023), and *SLC37A1* (*solute carrier family 37 member 1;* BTA1, Karamojong and Nganda10) implicated in fat metabolism and mammary gland development (Wang et al. 2022). *KHDRBS2 (KH RNA binding domain containing*,* signal transduction associated 2;* BTA23, Karamojong), has also been identified as a signature for adaptation to harsh environments in dual purpose cattle (Strillacci et al. 2020).

Taken together, these results underscore the role of genetic variation in the environmental adaptation of Ugandan indigenous cattle, enabling them to withstand heat, seasonal drought, nutritional deficits, and other ecological stressors characteristic of low-input pastoral and agro-pastoral production systems.

### Traits related to cattle temperament and farmer preferences

Traits related to handling and temperament play a significant role in livestock productivity and welfare, as well as behavior and several other traits (Haskell et al. 2014; Jaśkowski et al. 2023). Generally, less nervous animals are easier to manage, which reduces costs associated with handling equipment and labor requirements, while contributing to safer interactions between farm personnel and livestock. Beyond temperament, cattle keepers often prize animals for aesthetic or specific physical features. In African indigenous cattle, these include phenotypic traits such as coat color, rump length, body size and weight, tail and ear length, udder and teat conformation, scrotum size, back profile, and horn characteristics (Kabi et al. 2015; Masaba et al. 2024; Ndumu et al. 2008a, b). Other preferred traits extend to functional attributes such as disease resistance, reproductive efficiency, fertility, milk production, and early maturity (Kugonza et al. 2011).

Genes identified as putative signatures of selection within this category include the pseudogene *A0AAA9SS40_BOVIN* (ENSBTAG00000061529; BTA14, all populations), which has previously been mapped to QTL 282877 for visual score traits in Nellore cattle (Machado et al. 2022). *OSBPL6 (oxysterol binding protein like 6;* BTA2, identified in all populations except Ntuku) has been reported as a candidate gene influencing body height and size traits in yaks (Liu et al. 2023), while *FBXL7* (Nganda10) has been associated with morphological traits in Romanian Simmental cattle (Spătaru et al. 2025), as well as feet and leg conformation traits in Chinese Holstein (Abdalla et al. 2021).

In the Nganda10 population, several genes were identified, including *ASXL3(ASXL transcriptional regulator 3;* BTA24) and *ZZZ3* (*zinc finger ZZ-type containing 3;* BTA3). Both genes have been reported as selection signatures for horn development (Duarte et al. 2022; H. Wang et al. 2022). *CCDC178* (*coiled-coil domain containing 178;* BTA24) has been linked to body weight in Italian cattle (Mancin et al. 2022) and hoof disorders in Braunvieh and Fleckvieh cattle (Kosińska-Selbi et al. 2020), while *AGBL1* has been associated with conformation traits (Mandel et al. 2025). *LMNTD1* (*lamin tail domain containing 1;* BTA5) has been connected to stature in US Holsteins (Weller et al. 2018), and *PTPRO* has been linked to wither height and stature in beef and dairy cattle (Doyle et al. 2020). *GABRG1* has been implicated in emotional and behavioral control (Vani et al. 2025) and white spotting in cattle (Jivanji et al. 2019) and has been identified in ROH and selection signature analyses in Valdostana cattle (Mastrangelo et al. 2020).

In Ugandan indigenous cattle, behavior and temperament traits may be under selection for a variety of reasons. These include, but are not limited to, ease of handling during hand milking (Majalija et al. 2020; Miyama et al. 2020), harnessing draft power (Okello et al. 2015), efficient grazing and watering, long-distance movement in search of pasture and water, tolerance to smoking for vector control, and routine animal–human interactions (Wurzinger et al. 2008). In this study, the genes *ANKFN1* (*ankyrin repeat and fibronectin type III domain containing 1*; BTA19) and *FBXL2* (*F-box and leucine rich repeat protein 2*; BTA22), both under selection in Nganda10, together with *USP46* (ubiquitin specific peptidase 46; BTA6, identified in all populations) were enriched for the GO term GO:0001662 (behavioral fear response). This GO term, along with *USP46*, have been previously associated with temperament traits in indicine breeds (Dos Santos et al. 2017; Paredes-Sánchez et al. 2022; Shen et al. 2022). Additionally, QTL 66109 (duration of inactivity during open field test), which was previously mapped to BTA2 in an association study of behavior traits and milk production (Friedrich et al. 2016), was detected in Karamojong animals, and corresponded to *RAB3GAP1* (*RAB3 GTPase activating protein catalytic subunit 1*; BTA2). Similarly, *KHDRBS2*, also detected in this population, has been reported as a signature for temperament in Brahman and Yunling cattle (Shen et al. 2022). Other notable genes include *SPATA17 (spermatogenesis associated 17;* BTA16) and *SHANK2*, both identified in the Nganda10. *SPATA17* has been reported as selection signature for nervousness in crossbred *Bos indicus* cattle (Riley et al. 2016), while *SHANK2* has been associated with temperament traits in Nelore, Gir, and Red Sindhi cattle (Genuíno et al. 2025).

Notably, a few selection signatures were detected for morphological traits such as horn and coat patterns, despite the historical importance of these traits in traditional selection (Kabi et al. 2015; Kugonza et al. 2011; Ndumu et al. 2008a, b). This suggests a shift in breeding priorities towards nutrition and production traits, likely driven by population pressure on grazing lands, the impacts of climate change on pasture availability, and increasing demand for milk and meat. Moreover, urbanization, higher household incomes, and access to new markets have further reinforced this shift. Consequently, cattle keepers favor animals with traits for efficient feed and water use, faster growth, and higher productivity over those valued mainly for cosmetic features (Erdaw 2023; Komarek et al. 2021; Rege et al. 2011).

These findings suggest that farmer-driven trait preferences have likely contributed to the selection signals underlying behavioral and temperament traits in Ugandan indigenous cattle. Consistent selection for these traits over generations may have shaped the genomic architecture of these populations, highlighting the influence of human-mediated selection alongside natural environmental pressures. Understanding the genetic basis of farmer-preferred traits provides valuable insights for designing and improving breeding programs that align with local preferences while maintaining the adaptive potential and resilience of native cattle herds.

### Limitations

There are some limitations that should be considered when interpreting our findings. All of the studied cattle populations have been previously classified as *Bos indicus* yet the reference assembly (ARS-UCD1.3) used in this study is derived from a *Bos taurus* animal. In addition, annotations for the identified regions were based on the Ensembl annotation database. These factors may introduce bias in detecting genomic regions under putative selection, either through differences in read coverage and variant density or limitations of publicly available annotation data. In the analysis of runs of homozygosity, not all detected segments necessarily result from identity by descent (IBD), inbreeding, or selection and some may represent false positives detected by chance. Future research integrating multi-omics approaches and functional studies will be critical to validate and extend these findings.

Some of the regions under putative selection corresponded to novel genes in *Bos taurus* as annotated in Ensembl release 112 (*Bos taurus* ARS-UCD1.3). The status and annotation of these loci may change as genome assemblies and databases are updated. Consequently, some regions reported here as novel may later be confirmed as known coding or non-coding genes.

## Conclusion

This study provides the first comprehensive genome-wide investigation of putative selection signatures in six Ugandan indigenous cattle populations using two complementary approaches, RAiSD and ROH. Although each method detects different aspects of genomic variation, the two approaches collectively identified several candidate regions and genes of potential relevance to traits important in the local production systems. The detected signatures likely reflect the cumulative effects of farmers’ choices over generations to retain animals best suited to local production environments, management practices, and socio-cultural preferences, even when those decisions were informal or based on traditional knowledge.

Majority of the identified candidate genes overlapped with results reported in other cattle breeds, highlighting shared biological mechanisms underlying adaptation and productivity. Additionally, several candidate regions corresponded to Ensembl-annotated loci lacking functional characterization, provisionally labeled as novel genomic regions following Ensembl gene-status conventions. These findings underscore the need for enhanced genome annotation in cattle, especially for indigenous African breeds that may harbor important, yet uncharacterized loci relevant to future breeding programs.

Overall, the results from this study contribute valuable genomic insights into Ugandan cattle populations and establish a foundation for future research focused on functional validation, improved breed characterization, and the integration of genomic information into sustainable breeding and conservation programs for indigenous livestock in tropical production environments.

## Supplementary Information

Below is the link to the electronic supplementary material.

Supplementary material 1 (XLSX 164.0 kb)

Supplementary material 2 (PDF 160.7 kb)

## Data Availability

The raw sequence data analyzed in this study is available in the European Nucleotide Archive (ENA) under the project accession number PRJEB90914.
