# Supplementary material for: Detection of selection signatures in indigenous African cattle reveals genomic footprints of adaptation, production and temperament traits
Source: Mamm Genome. 2026 Jan 28;37(1):27. doi: 10.1007/s00335-026-10193-9 (PMC12852251; doi:10.1007/s00335-026-10193-9)
Supplement: Supplementary file 2 — Supplementary material 2 (PDF 160.7 kb) [file 335_2026_10193_MOESM2_ESM.pdf]

Table SF1: The modified (and default) PLINK parameters for this study

Description in text	PLINK tag	Default	This study
**Sliding window size**	--homozyg-window-snp	100	50
**Heterozygous calls per window**	--homozyg-window-het	1	2
**Missing calls per window**	--homozyg-window-missing	5	10
**Homozygous windows**	--homozyg-window-threshold	0.05	0.025
**Minimum ROH length**	--homozyg-kb	1000	500
**Minimum SNPs per ROH**	--homozyg-snp	100	50
**Minimum SNP density**	--homozyg-density	50	500

To ensure that downstream inferences were not driven by parameter choice, the ROH calling results with PLINK's default ROH detection parameters and the WGS-optimized ROH parameters (Table SF1) were compared, and the concordance between the two parameterizations assessed.

Table SF2: Sensitivity of ROH detection with default and WGS-optimized parameter settings

Population	ROH class	PLINK default ROH parameters		PLINK ROH parameters used in this study	
		mean nROH	mean size (kb)	mean nROH	mean size (kb)
**Ankole**	0.5–1 Mb	-	-	2369	1579955.760
	1–2 Mb	319	408465.139	483	625525.079
	2–4 Mb	43	104169.02	72	185717.953
	>4 Mb	1	5952.349	14	82716.172
**Karamojong**	0.5–1 Mb	-	-	1029	677930.447
	1–2 Mb	52	66487.012	125	153654.944
	2–4 Mb	4	9898.831	10	23335.292
	>4 Mb	-	-	2	9508.169
**Nganda10**	0.5–1 Mb	-	-	1488	1018388.803
	1–2 Mb	6	7263.917	425	550458.307
	2–4 Mb	-	-	36	87390.014
	>4 Mb	-	-	1	4235.150
**Nganda17**	0.5–1 Mb	-	-	1707	1132273.082
	1–2 Mb	342	451234.485	381	512092.884
	2–4 Mb	78	202656.28	100	274722.089
	>4 Mb	6	29030.619	44	259366.702

**Nkedi**	0.5–1 Mb	-	-	1844	1232009.376
	1–2 Mb	372	494016.985	434	563136.944
	2–4 Mb	77	197142.266	113	307911.759
	>4 Mb	7	30130.922	49	297652.248
**Ntuku**	0.5–1 Mb	-	-	2203	1467419.437
	1–2 Mb	472	622217.585	549	724417.322
	2–4 Mb	99	259525.567	157	434324.753
	>4 Mb	13	62039.555	55	319369.053

Comparison of the number and total length of runs of homozygosity (ROH) detected using PLINK's default parameters and WGS-optimized parameters. WGS-optimized settings increased sensitivity to shorter ROH segments (<1 Mb) (Table SF2).

Table SF3: The F_{ROH} estimates and mean number (nROH) obtained using default PLINK parameters and WGS-optimized parameters

**Population**	***n***	**PLINK default ROH parameters**			**PLINK ROH parameters used in this study**		
		mean F_{ROH}	SD F_{ROH}	mean nROH	mean F_{ROH}	SD F_{ROH}	mean nROH
**Ankole**	19	0.0110	0.0063	19.1053	0.0523	0.0140	154.6316
**Karamojong**	11	0.0028	0.0016	5.0909	0.0316	0.0043	106.0000
**Nganda10**	10	0.0003	0.0004	0.6000	0.0667	0.0693	195.0000
**Nganda17**	17	0.0161	0.0199	25.0588	0.0515	0.0278	131.2941
**Nkedi**	19	0.0152	0.0400	24.0000	0.0508	0.0581	128.4211
**Ntuku**	19	0.0200	0.0163	30.7368	0.0623	0.0240	156.0000

The F_{ROH} estimates (Table SF3) obtained using WGS-optimized ROH parameters were highly concordant with those derived using PLINK's default settings. Individual-level F_{ROH} values showed strong rank and linear agreement between parameterizations (Spearman $\rho = 0.75$; Pearson $r = 0.77$; both $p < 10^{-18}$), indicating that relative patterns of autozygosity were preserved despite differences in absolute ROH calling thresholds.

Given higher marker density and uneven coverage associated with whole-genome sequencing data, we therefore adopted results derived with WGS-optimized ROH parameters for downstream analyses.
